# Supplementary material for: Epidemiology and Future Burden of Vertebral Fractures: Insights from the Global Burden of Disease 1990–2021
Source: Healthcare (Basel). 2025 Jul 22;13(15):1774. doi: 10.3390/healthcare13151774 (PMC12346102; doi:10.3390/healthcare13151774)
Supplement: Supplementary file 1 [file healthcare-13-01774-s001.zip › healthcare-3598470-supplementary.pdf]

	Number of prevalent cases	Age-standardized prevalence rate per 100 000	Percentage change in age-standardized prevalence rate from 1990 to 2021	Number of YLDs	Age-standardized rate of YLDs per 100 000	Percentage change in age-standardized rate of YLDs per 100,000 from 1990 to 2021
Global	1016688 (813537 to 1285026)	29 (23 to 37)	-11.28% (-10.93 to -11.04%)	107946 (69844 to 158795)	3 (2 to 5)	-11.89% (-10.71 to -12.73%)
Central Europe, Eastern Europe, and Central Asia	66584 (52083 to 85771)	37 (29 to 48)	-14.99% (-14.14 to -15.36%)	7088 (4382 to 10645)	4 (2 to 6)	-15.3% (-14.44 to -15.46%)
Central Asia	8944 (6849 to 11741)	20 (15 to 26)	-11.04% (-9.1 to -11.72%)	963 (567 to 1511)	2 (1 to 3)	-11.6% (-12.85 to -11.13%)
Central Europe	18199 (13974 to 24043)	40 (30 to 52)	-12.76% (-11.91 to -13.02%)	1941 (1172 to 2978)	4 (3 to 6)	-13.21% (-12.94 to -13.31%)
Eastern Europe	39442 (31187 to 50359)	45 (35 to 57)	-10.11% (-8.92 to -10.4%)	4184 (2577 to 6287)	5 (3 to 7)	-10.42% (-9.34 to -10.6%)
High-income	413110 (343612 to 503878)	95 (79 to 115)	2.46% (1.78 to 3.38%)	43245 (28010 to 62713)	10 (6 to 14)	1.61% (1.34 to 0.65%)
Australasia	13682 (11215 to 16915)	106 (87 to 131)	-4.13% (-3.92 to -4.1%)	1441 (842 to 2236)	11 (7 to 17)	-4.45% (-5.55 to -4.47%)
High-income Asia Pacific	52449 (43985 to 63547)	77 (64 to 93)	-14.44% (-14.72 to -13.98%)	5549 (3504 to 8250)	8 (5 to 12)	-14.85% (-15.06 to -14.41%)
High-income North America	160613 (132572 to 195887)	105 (87 to 128)	15.82% (14.61 to 16.18%)	16685 (10687 to 24354)	11 (7 to 16)	14.27% (14.15 to 13.62%)
Southern Latin America	166327 (135993 to 205887)	96 (79 to 119)	-2.6% (-3.97 to -1.19%)	17453 (11072 to 25708)	10 (6 to 15)	-2.98% (-3.18 to -3.5%)
Western Europe	55826 (42451 to 73738)	20 (16 to 27)	-11.11% (-6.73 to -13.5%)	5992 (3703 to 9102)	2 (1 to 3)	-12% (-8.97 to -13.86%)
Latin America and Caribbean	4689 (3534 to 6237)	15 (12 to 21)	-3.74% (-1.01 to -6.86%)	508 (287 to 827)	2 (1 to 3)	-4.38% (-7.63 to -2.84%)
Andean Latin America	4428 (3200 to 6462)	21 (15 to 30)	48.46% (41.22 to 64.43%)	472 (265 to 777)	2 (1 to 4)	45.16% (38.94 to 53.45%)
Caribbean	23463 (17596 to 31605)	20 (15 to 27)	-20.1% (-16.43 to -22.13%)	2526 (1515 to 3916)	2 (1 to 3)	-20.84% (-19.72 to -21.25%)
Central Latin America	23247 (17610 to 30964)	22 (17 to 30)	-9.1% (-4.77 to -12.42%)	2486 (1479 to 3835)	2 (1 to 4)	-9.96% (-7.51 to -12.24%)
Tropical Latin America	67503 (50206 to 92907)	24 (18 to 33)	-4.27% (-3.4 to -3.32%)	7198 (4459 to 11003)	3 (2 to 4)	-5.64% (-4.51 to -7.67%)
North Africa and Middle East	161839 (120284 to 218197)	19 (14 to 26)	0.11% (3.01 to -2.15%)	17373 (10634 to 26544)	2 (1 to 3)	-0.63% (3.26 to -2.13%)
South Asia	196852 (155175 to 253123)	21 (17 to 27)	16.16% (19.66 to 14.64%)	21088 (13300 to 31432)	2 (1 to 3)	14.89% (18.47 to 12.99%)
Southeast Asia, East Asia, and Oceania	147835 (116632 to 191327)	24 (19 to 31)	27.92% (31.4 to 26.18%)	15796 (9789 to 23785)	3 (2 to 4)	26.33% (29.21 to 24.71%)
East Asia	1299 (959 to 1762)	20 (15 to 27)	33.66% (35.48 to 32.6%)	140 (77 to 231)	2 (1 to 4)	33.11% (30.28 to 35.94%)
Oceania	47718 (36794 to 61837)	15 (11 to 19)	-7.1% (-3.86 to -10.67%)	5151 (3240 to 7838)	2 (1 to 2)	-7.72% (-4.29 to -8.8%)
Southeast Asia	54974 (40004 to 77279)	10 (7 to 14)	-28.36% (-25.73 to -31.33%)	5962 (3660 to 9176)	1 (1 to 2)	-28.67% (-26.38 to -32.1%)
Sub-Saharan Africa	7321 (5337 to 10199)	11 (8 to 15)	-13.97% (-14.64 to -12.3%)	792 (450 to 1274)	1 (1 to 2)	-14.38% (-15.13 to -14.82%)
Central Sub-Saharan Africa	21126 (14491 to 32602)	10 (7 to 16)	-39.82% (-34.41 to -44.71%)	2280 (1356 to 3663)	1 (1 to 2)	-40.37% (-34.54 to -46.51%)
Eastern Sub-Saharan Africa	4425 (3493 to 5672)	12 (9 to 15)	-31.98% (-29.67 to -32.91%)	474 (285 to 727)	1 (1 to 2)	-32.43% (-32.22 to -32.92%)
Southern Sub-Saharan Africa	22101 (16267 to 30016)	9 (7 to 12)	-10.45% (-9.14 to -11.97%)	2416 (1474 to 3723)	1 (1 to 2)	-10.61% (-7.74 to -11.55%)
Western Sub-Saharan Africa	1016688 (813537 to 1285026)	29 (23 to 37)	-11.28% (-10.93 to -11.04%)	107946 (69844 to 158795)	3 (2 to 5)	-11.89% (-10.71 to -12.73%)

Table S1. Global prevalence, YLDs, and age-standardized rates of prevalence and YLDs per 100 000 population in 2021, and percentage change between 1990 and 2021 for fractures of the vertebral column among women aged under 65 years, by GBD regions and super-regions.

	Number of prevalent cases	Age-standardized prevalence rate per 100 000	Percentage change in age-standardized prevalence rate from 1990 to 2021	Number of YLDs	Age-standardized rate of YLDs per 100 000	Percentage change in age-standardized rate of YLDs per 100,000 from 1990 to 2021
Global	1660320 (1342944 to 2074981)	394 (319 to 493)	-3.74% (-4.79 to -2.53%)	159717 (102395 to 229921)	38 (24 to 55)	-4.75% (-5.58 to -3.81%)
Central Europe, Eastern Europe, and Central Asia	68127 (58049 to 80754)	175 (149 to 208)	-13.72% (-14.06 to -13.19%)	6637 (4396 to 9310)	17 (11 to 24)	-14.16% (-14.34 to -15%)
Central Asia	2786 (2463 to 3196)	78 (69 to 89)	-5.53% (-5.98 to -5.65%)	274 (179 to 401)	8 (5 to 11)	-6.17% (-4.69 to -3.61%)
Central Europe	33124 (26966 to 40868)	250 (204 to 309)	-23.81% (-22.48 to -24.81%)	3226 (2080 to 4626)	24 (16 to 35)	-23.96% (-23.89 to -25.01%)
Eastern Europe	32217 (28115 to 37173)	146 (128 to 169)	-9.09% (-10.42 to -7.4%)	3137 (2095 to 4392)	14 (10 to 20)	-9.82% (-10.16 to -10.54%)
High-income	1112031 (887302 to 1402244)	936 (747 to 1181)	11.56% (10.4 to 12.79%)	106476 (67716 to 154293)	90 (57 to 130)	10.13% (9.45 to 11.79%)
Australasia	32517 (25597 to 42130)	1175 (925 to 1523)	29.46% (26.42 to 34.19%)	3117 (1957 to 4598)	113 (71 to 166)	27.82% (25.9 to 29.13%)
High-income Asia Pacific	145843 (120799 to 178166)	563 (466 to 687)	1.32% (-1.22 to 5.07%)	14158 (9145 to 20125)	55 (35 to 78)	0.09% (-1.54 to 2.14%)
High-income North America	371652 (295503 to 466379)	1052 (836 to 1320)	38.92% (35.69 to 40.94%)	35324 (22664 to 50701)	100 (64 to 143)	36.38% (35.23 to 38.03%)
Southern Latin America	539136 (423690 to 691005)	1077 (846 to 1380)	7.4% (6.45 to 8.68%)	51656 (32406 to 75835)	103 (65 to 151)	6.29% (5.32 to 8.22%)
Western Europe	44668 (37255 to 54141)	152 (126 to 184)	0.38% (-0.74 to 1.68%)	4340 (2835 to 6208)	15 (10 to 21)	-0.43% (-1.61 to -0.17%)
Latin America and Caribbean	2492 (2130 to 2932)	94 (80 to 110)	16.37% (12.9 to 20.22%)	244 (151 to 366)	9 (6 to 14)	15.44% (14.12 to 14.42%)
Andean Latin America	6247 (4953 to 8009)	242 (192 to 310)	65.43% (63.64 to 69.58%)	606 (378 to 889)	23 (15 to 34)	61.51% (61.26 to 58.05%)
Caribbean	14903 (12499 to 17939)	128 (107 to 154)	-23.52% (-23.46 to -23.72%)	1454 (951 to 2093)	12 (8 to 18)	-23.76% (-25.32 to -23.18%)
Central Latin America	21026 (17247 to 25807)	167 (137 to 205)	8.87% (5.66 to 11.79%)	2036 (1293 to 2954)	16 (10 to 23)	8.08% (6.62 to 9.79%)
Tropical Latin America	20336 (17058 to 24655)	118 (99 to 143)	19.3% (14.93 to 22.96%)	1980 (1317 to 2835)	12 (8 to 16)	18.12% (16.96 to 18.31%)
North Africa and Middle East	170447 (130784 to 220642)	275 (211 to 356)	17.14% (17.31 to 15.57%)	16410 (10363 to 24343)	26 (17 to 39)	16.72% (17.65 to 16.75%)
South Asia	228565 (187532 to 282081)	168 (138 to 207)	38.15% (33.56 to 43.2%)	22285 (14420 to 32092)	16 (11 to 24)	36.23% (33.71 to 38.04%)
Southeast Asia, East Asia, and Oceania	189872 (154978 to 234681)	176 (144 to 218)	46.13% (40.23 to 52.22%)	18509 (11908 to 26761)	17 (11 to 25)	43.82% (41.09 to 46.36%)
East Asia	391 (316 to 486)	166 (134 to 206)	52.12% (48.09 to 55.32%)	38 (23 to 58)	16 (10 to 24)	50.47% (55.38 to 49.09%)
Oceania	38301 (31547 to 47133)	134 (111 to 165)	8.74% (6.74 to 10.33%)	3738 (2423 to 5418)	13 (9 to 19)	7.95% (7.19 to 7.48%)
Southeast Asia	16146 (13582 to 19451)	88 (74 to 106)	5.08% (3.48 to 7.09%)	1590 (1065 to 2255)	9 (6 to 12)	5.15% (4.16 to 5.66%)
Sub-Saharan Africa	1704 (1447 to 2050)	84 (72 to 101)	18.75% (16.46 to 22.62%)	170 (100 to 259)	8 (5 to 13)	20.25% (21.25 to 18.84%)
Central Sub-Saharan Africa	5842 (4837 to 7211)	92 (76 to 113)	7.75% (5.84 to 11.04%)	574 (383 to 832)	9 (6 to 13)	7.07% (6.2 to 8.81%)
Eastern Sub-Saharan Africa	1562 (1379 to 1781)	57 (50 to 65)	-25.37% (-26.12 to -24.54%)	152 (100 to 216)	6 (4 to 8)	-26.08% (-25.87 to -25.5%)
Southern Sub-Saharan Africa	7037 (5832 to 8584)	98 (81 to 119)	10.53% (9.17 to 11.6%)	695 (450 to 997)	10 (6 to 14)	11.12% (8.55 to 10.56%)
Western Sub-Saharan Africa	1660320 (1342944 to 2074981)	394 (319 to 493)	-3.74% (-4.79 to -2.53%)	159717 (102395 to 229921)	38 (24 to 55)	-4.75% (-5.58 to -3.81%)

Table S2. Global prevalence, YLDs, and age-standardized rates of prevalence and YLDs per 100 000 population in 2021, and percentage change between 1990 and 2021 for fractures of the vertebral column among women aged 65 years and older, by GBD regions and super-regions.

	Number of prevalent cases	Age-standardized prevalence rate per 100 000	Percentage change in age-standardized prevalence rate from 1990 to 2021	Number of YLDs	Age-standardized rate of YLDs per 100 000	Percentage change in age-standardized rate of YLDs per 100,000 from 1990 to 2021
(A) Women						
Global	5,371,438 (4,703,837 to 6,196,132)'	65 (57 to 75)	–0.21% (–0.22 to –0.19)	545,923 (366,571 to 757,099)	65 (57 to 75)	–0.21% (–0.22 to –0.19)
Low SDI	246714 (190571 to 325486)	33 (28 to 41)	-0.01% (0 to -0.05)	26118 (17629 to 38250)	3 (2 to 5)	-0.02% (0 to -0.04)
Low-middle SDI	567486 (471693 to 669476)	37 (31 to 43)	-0.05% (-0.03 to -0.05)	59226 (40462 to 84059)	4 (3 to 5)	-0.06% (-0.06 to -0.06)
Middle SDI	996724 (846944 to 1163380)	40 (34 to 46)	0% (0 to 0)	103474 (69771 to 145983)	4 (3 to 6)	-0.01% (-0.01 to -0.02)
High-middle SDI	1086362 (953352 to 1244962)	64 (56 to 75)	-0.23% (-0.23 to -0.22)	111095 (75248 to 154768)	7 (4 to 9)	-0.23% (-0.22 to -0.21)
High SDI	2469882 (2144431 to 2817433)	132 (115 to 149)	-0.16% (-0.17 to -0.15)	245574 (163359 to 339966)	13 (9 to 19)	-0.16% (-0.17 to -0.16)
(B) Women aged under 65 years						
Global	5,371,438 (4,703,837 to 6,196,132)'	65 (57 to 75)	–0.21% (–0.22 to –0.19)	545,923 (366,571 to 757,099)	65 (57 to 75)	–0.21% (–0.22 to –0.19)
Low SDI	396127 (279205 to 573752)	17 (12 to 25)	-14.51% (-12.68 to -16.02%)	42688 (26452 to 66939)	2 (1 to 3)	-14.92% (-10.75 to -17.6%)
Low-middle SDI	757489 (580517 to 981854)	20 (16 to 26)	-7.12% (-3.52 to -10.15%)	81694 (51404 to 122048)	2 (1 to 3)	-7.61% (-4.56 to -9.94%)
Middle SDI	1235551 (968527 to 1579183)	26 (21 to 33)	4.67% (9.05 to 2.17%)	132683 (84996 to 195736)	3 (2 to 4)	3.61% (7.31 to 0.94%)
High-middle SDI	1118549 (909717 to 1388947)	47 (39 to 59)	-8.07% (-6.57 to -8.67%)	119415 (76582 to 174639)	5 (3 to 7)	-8.38% (-7.17 to -9.29%)
High SDI	1730763 (1452223 to 2091134)	95 (80 to 115)	-8.85% (-9.1 to -8.3%)	182408 (119081 to 262043)	10 (7 to 14)	-9.59% (-9.62 to -10.21%)
(C) Women aged 65 years and older						
Global	97301 (80698 to 118485)	130 (107 to 159)	17.37% (15.41 to 18.66%)	9547 (6427 to 13557)	13 (9 to 18)	17.47% (17.21 to 16.97%)
Low SDI	377482 (310447 to 461126)	160 (131 to 198)	10.79% (9.98 to 11.21%)	36758 (24215 to 52620)	16 (10 to 22)	10.46% (10.61 to 10.52%)
Low-middle SDI	757897 (634306 to 915904)	163 (136 to 197)	28.09% (24.32 to 32.21%)	74266 (48868 to 105158)	16 (11 to 23)	27.14% (25.63 to 27.88%)
Middle SDI	1054175 (888381 to 1263580)	285 (240 to 341)	-13.11% (-13.77 to -11.94%)	102774 (67452 to 144723)	28 (18 to 39)	-13.5% (-13.99 to -12.68%)
High-middle SDI	3209000 (2605291 to 3968029)	781 (633 to 968)	8.07% (5.91 to 10.18%)	308741 (199309 to 441077)	75 (48 to 108)	6.85% (6.15 to 8.73%)
High SDI	97301 (80698 to 118485)	130 (107 to 159)	17.37% (15.41 to 18.66%)	9547 (6427 to 13557)	13 (9 to 18)	17.47% (17.21 to 16.97%)

Table S3. Global prevalence, YLDs, age-standardized prevalence and YLDs rates per 100 000 population in 2021, and percentage change between 1990 and 2021 for fracture of the vertebral column among (A) women, (B) women aged under 65 years, (C) Women aged 65 years and older, by Socio-demographic Index
